# Supplementary material for: Identification of an additive interaction using parameter regularization and model selection in epidemiology
Source: PeerJ. 2024 Oct 14;12:e18304. doi: 10.7717/peerj.18304 (PMC11485060; doi:10.7717/peerj.18304)
Supplement: Supplemental Information 3 [file peerj-12-18304-s003.docx]

Table S1 Hypertension example^a^

|  |  | Hypertension | |
| --- | --- | --- | --- |
| age | BMI | Yes | No |
| 1 | 1 | 278 | 743 |
| 1 | 0 | 100 | 581 |
| 0 | 1 | 153 | 1232 |
| 0 | 0 | 79 | 1731 |

^a^ data was from the example presented by Guang Yong Zou (Zou, 2008)

Table S2 The coefficients and GDEV of the hypertension example

| model | $\hat{\theta}_{1}$ | $\hat{\theta}_{2}$ | $\hat{\theta}_{3}$ | GDEV |
| --- | --- | --- | --- | --- |
| $M_{u}$ | 1.327 | 1.000 | 2.104 | 3375.70 |
| $M_{c}$ | 1.638 | 1.164 | 1.99 | 3388.79 |

Table S3 Congenital heart defect example^a^

|  |  | Secondhand Smoke | |
| --- | --- | --- | --- |
| Household Per Capita Monthly Income | Congenital Heart Defect | Yes | No |
| ≤2000 | Yes | 425 | 1863 |
| ≤2000 | No | 210 | 1773 |
| ＞2000 | Yes | 598 | 1840 |
| ＞2000 | No | 522 | 2221 |

^a^ data was from the example presented by Zhi Qiang Nie (Nie et al., 2016)

Table S4 The coefficients and GDEV of the Congenital heart defect example

| model | $\hat{\theta}_{1}$ | $\hat{\theta}_{2}$ | $\hat{\theta}_{3}$ | GDEV |
| --- | --- | --- | --- | --- |
| $M_{u}$ | 0.238 | 0.324 | 0.893 | 12985.81 |
| $M_{c}$ | 0.287 | 0.459 | 0.650 | 13000.69 |
